# 

**Published:** 1887-02

**Authors:** 


					﻿no cftt/a copy.
FEBRUARY, 1887.
$1.00 per year.
HALES «
@JOV1\N.AL-J
healt h
ESTABLISHED 185 4
Vi-34*
2.
OFFICE: 206 BBOADWAY. EVENING POST BUILDING. Boom 11. N. Y.
				

